# Loss of Smad7 Promotes Inflammation in Rheumatoid Arthritis

**DOI:** 10.3389/fimmu.2018.02537

**Published:** 2018-11-02

**Authors:** Gengmin Zhou, Xiaolin Sun, Qingxia Qin, Jiyang Lv, Yueming Cai, Meiying Wang, Rong Mu, Hui-yao Lan, Qing-Wen Wang

**Affiliations:** ^1^Department of Rheumatism and Immunology, Peking University Shenzhen Hospital, Shenzhen, China; ^2^Department of Rheumatology and Immunology, Peking University People's Hospital, Peking, China; ^3^Department of Medicine and therapeutics, Li KaShing Institute of Health Sciences, The Chinese University of Hong Kong, Shatin, China

**Keywords:** SMAD7, RA, Th17 & Tregs cells, TGF-beta 1, inflammation immunomodulation

## Abstract

**Objective:** Smad7 is an inhibitory Smad and plays a protective role in many inflammatory diseases. However, the roles of Smad7 in rheumatoid arthritis (RA) remain unexplored, which were investigated in this study.

**Methods:** The activation of TGF-β/Smad signaling was examined in synovial tissues of patients with RA. The functional roles and mechanisms of Smad7 in RA were determined in a mouse model of collagen-induced arthritis (CIA) in Smad7 wild-type (WT) and knockout (KO) CD-1 mice, a strain resistant to autoimmune arthritis induction.

**Results:** TGF-β/Smad3 signaling was markedly activated in synovial tissues of patients with RA, which was associated with the loss of Smad7, and enhanced Th17 and Th1 immune response. The potential roles of Smad7 in RA were further investigated in a mouse model of CIA in Smad7 WT/KO CD-1 mice. As expected, Smad7-WT CD-1 mice did not develop CIA. Surprisingly, CD-1 mice with Smad7 deficiency developed severe arthritis including severe joint swelling, synovial hyperplasia, cartilage damage, massive infiltration of CD3^+^ T cells and F4/80^+^ macrophages, and upregulation of proinflammatory cytokines IL-1β, TNFα, and MCP-1. Further studies revealed that enhanced arthritis in Smad7 KO CD-1 mice was associated with increased Th1, Th2 and, importantly, Th17 over the Treg immune response with overactive TGF-β/Smad3 and proinflammatory IL-6 signaling in the joint tissues.

**Conclusions:** Smad7 deficiency increases the susceptibility to autoimmune arthritis in CD-1 mice. Enhanced TGF-β/Smad3-IL-6 signaling and Th17 immune response may be a mechanism through which disrupted Smad7 causes autoimmune arthritis in CD-1 mice.

## Introduction

Rheumatoid arthritis (RA) is an autoimmune disease with chronic inflammation, synovial hyperplasia, and joint destruction, deformity and loss of function. RA synovitis shows massive leukocyte infiltration, proliferative synovial cells and pannus formation, which are responsible for the cartilage and bone destruction (1). Previous studies have demonstrated that inflammatory cytokines and chemokines play an important role in the pathogenesis of RA (2–4). Transforming growth factor-β (TGF-β) and nuclear factor kappa-B (NF-κB) signaling are crucial regulatory pathways in RA (5, 6, 7). It is well accepted that the biological activities of TGF-β are mediated through both type I and type II receptors to regulate TGF-β-responsive genes by inducing phosphorylation of two downstream signaling molecules Smad2 and Smad3 (8, 9). In addition, TGF-β also induces Smad7 expression, an inhibitory Smad family member, to negatively regulate TGF-β/Smad signaling via a feedback mechanism (10, 11). However, the regulatory roles of Smad7 in inflammation and autoimmunity are still controversial. It has been shown that disrupted Smad7 promotes renal inflammation and fibrosis via both TGF-β/Smad and NF-κB signaling pathways, which are inhibited by overexpressing renal Smad7 (11–16). It is also reported that Smad7 deficiency increases TGF-β signaling and inhibits T cell activation, while the overexpression of Smad7 enhances the resistance of autoimmune T cells to Treg suppression (17–19). In autoimmune arthritis such as RA, the exact functions of Smad7 remain unexplored. Thus, this study examined the potential roles of Smad7 in synovial tissues from RA patients and in a mouse model of collagen-induced arthritis (CIA) in Smad7 KO CD-1 mice.

## Research design and methods

### Patients

Synovial tissues were obtained from a total of 14 patients. All patients fulfilled the 1987 criteria of rheumatoid arthritis of the American College of Rheumatology (20). Clinically, these RA patients (5 male and 9 female) had the mean age of 50.2 ± 16.8 years and disease duration over 73.8 ± 49.6 months and developed high levels of Disease Activity Score-28 (4.9 ± 0.9), erythrocyte sedimentation rate (64.3 ± 21.9 mm/h), and hs-CRP (24.1 ± 19.3 mg/L). Control synovial tissue specimens were obtained from 5 traumatic patients with knee tear ligament surgery without any evidence of rheumatoid disease (2 males and 3 females; mean age at 41.8 ± 13.5 years). All samples were fixed in formalin for paraffin section or in liquid nitrogen for frozen section. All patients were informed about the aims of specimen collection and given the signed written consent. The study was approved and performed in accordance with the ethical guidelines of Peking University Shenzhen Hospital.

### Animal models and clinical parameters

Smad7 knockout (KO) and wild-type (WT) CD-1 male mice were used in this study. Smad7 KO mice were generated by functionally deleting exon I in the Smad7 gene as previously described (21). The genotype of Smad7 KO mice was confirmed by PCR with the primers (21). It is well known that the CD-1 mice are an outbred stock. To overcomethe genetic heterogeneity of outbred CD-1 mice, all Smad7 KO/WT mice were inbred for at least 20 consecutive generations to obtain their homogenous genetic background. This CD-1 inbred strain was used in this study. We adopted the well-established method of collagen-induced arthritis (CIA) (22, 23) in genetically identical littermate Smad7 KO and WT CD-1mice (*n* = 8/group, male, aged 8–10 weeks, 32.29 ± 3.2 g) by intracutaneous injection of the mixture of 100 μl chicken collagen II (5 mg/ml, Sigma, St. Louis, MO, United States) emulsified with the complete Freund's adjuvant (CFA, 4 mg/ml, Sigma) at the base of the tail. Fourteen days later, these mice received the second immunization of the mixture of collagen II and the incomplete Freund's adjuvant. Control mice followed the same protocol except they received saline only. In addition, group of 6 normal Smad7 WT/KO mice at the same age were used as normal control. All mice were sacrificed at 10 weeks for collecting blood, synovium and joints for disease evaluation. The clinical severity of arthritis was assessed as previously described (24): (0) normal without detectable lesions; (0.5) erythema + edema in only one digit; (1) erythema + mild edema detectable in the footpad or ankle, or two to five digits; (2) erythema + moderate edema detectable in two joints (footpad, ankle), or two to five digits; (3) erythema + severe edema of the entire paw; (4) reduced swelling but deformation with incapacitated limb. Individual score was obtained by two investigators who were unaware of the mouse identity, and the mean value was calculated. All experimental procedures were approved by the Animal Experimentation Etheric Committee at the Chinese University of Hong Kong.

### Real-time PCR

Synovium tissues were collected by carefully removing the bilateral knee joint and they were kept at −80°C freezer before being analyzed of the genes of interest using quantitative real-time PCR as previously described (16). The primers used in this study including tumor necrosis factor-α (TNF-α), interleukin-1β (IL-1β), TGF-β, and the house keeping gene GAPDH as described previously (16), whereas primers for interleukin-6 (IL-6), RORγt,interleukin-17A (IL-17A), Foxp3, interleukin-10(IL-10), T-bet and GATA-3were described below:

IL-6 forward:5-AGGATACCACTCCCAACAGACCT-3; reverse:5-CAAGTGCATCATCGTTGTTCATAC-3;

RORγt forward:5-CCGCTGAGAGGGCTTCAC-3; reverse:5-TGCAGGAGTAGGCCACATTACA-3;

IL-17A forward:5-TTTAACTCCCTTGGCGCAAAA-3; reverse:5- CTTTCCCTCCGCATTGACAC-3;

Foxp3 forward: 5-GCACCTTCCCAAATCCCAGT-3; reverse: 5-GGCCACTTGCAGACACCAT-3;

T-bet forward: 5-CGGCTGCATATCGTTGAGGT-3; reverse: 5-GTCCCCATTGGCATTCCTC-3;

GATA-3forward: 5-ACCGGCTTCGGATGCAA-3; reverse: 5-GCCTTCGCTTGGGCTTAAT-3.

House keeping gene GAPDH was used as an internal standard. The ratios of the mRNAs examined against GAPDH were obtained and expressed as mean ± S.E.

### Elisa

The ELISA Kit for IL-17A was purchased from R&D (Minneapolis, MN, United States) and ELISA kits for TNF-α, IL-1β and TGF-β were obtained from Santa Cruz (California, USA). Plasma levels of TNF-α, IL-1β, TGF-β and IL-17A were detected by ELISA according to the manufacturer's protocol. In addition, serum levels of mouse anti-collagen II IgG and subclasses of IgG1 and IgG2a were also measured by ELISA using the ELISA kits obtained from Chondrex, Inc. (Redmond, WA, United States).

### Histology and immunohistochemistry

The pathological changes in synovial tissues and joints were examined in paraffin-embedded tissue sections (4 μm)by hematoxylin-eosin (HE) staining. Immunohistochemistry (IHC) was performed on paraffin sections using the microwave-based antigen retrieval technique. The antibodies used in this study were as followed: CD3, IL-17, IL-6 and Foxp3 (Abcam, Cambridge, United Kingdom), TNF-α, IL-1β, TGF-β, TGF-β receptor II, Smad7 (Santa Cruz, California, United States), F4/80 (Serota, Raleigh, North Carolina, United States), MCP-1 (eBiosience, San Diego, CA, United States),phospho-Smad3 (Rockland, Philadelphia, United States), phospho-p65 (Cell signaling, Beverly, MA, United States), rabbit anti-rat secondary antibody, rabbit anti-goat secondary antibody and anti-rabbit polymer (DAKO, Carpinteria, CA, United States). Expression levels of TGF-β,TGF-β receptor II, Smad7, TNF-α, IL-1β, MCP-1, IL-17, and IL-6 in synovial tissues were analyzed and determined using the quantitative Image Analysis System (AxioVision 4, Carl Zeiss, Germany) as previously described (1). The number of cells positive for phopsho-p65, phospho-Smad3, T-bet, Gata3, CD3, andF4/80 were counted in 5 consecutive high power fields (40x) by means of a 0.0625-mm^2^ graticule fitted in the eyepiece of the microscope and expressed as cells per millimeters squared.

### Immunofluorescence

Two-color immunofluorescence (IF) was performed on frozen sections with PE-labeled rat anti-mouse CD4, FITC-labeled rat anti-mouse IL-17A, FITC-labeled rat anti-mouse Foxp3, FITC-labeled rat anti-mouse IFN-γ and FITC-labeled rat anti-mouseGata3 (eBioscience, San Diego, CA, United States). Single, double, or triple positive cells were counted under fluorescent microscope (Axioplan2 imaging, Carl Zeiss, Oberkoche, Germany) in 10 consecutive high-power fields (40x) per section by means of a 0.0625 mm^2^ graticule fitted in the eyepiece of the microscope and expressed as cells per mm^2^.

### Statistical analysis

Data were presented as the mean ± S.E. and statistical significance between groups was assessed with one-way analysis of variance (ANOVA) or two-way ANOVA, followed by Tukey's *post-hoc* test using the Prism 5.0 GraphPad Software (San Diego, CA, United States).

## Results

### Smad7 is largely reduced in synovial tissues of patients with RA, which is associated with enhanced TGF-β/Smad3 signaling and Th17 and Th1 responses

**Figure 1:**
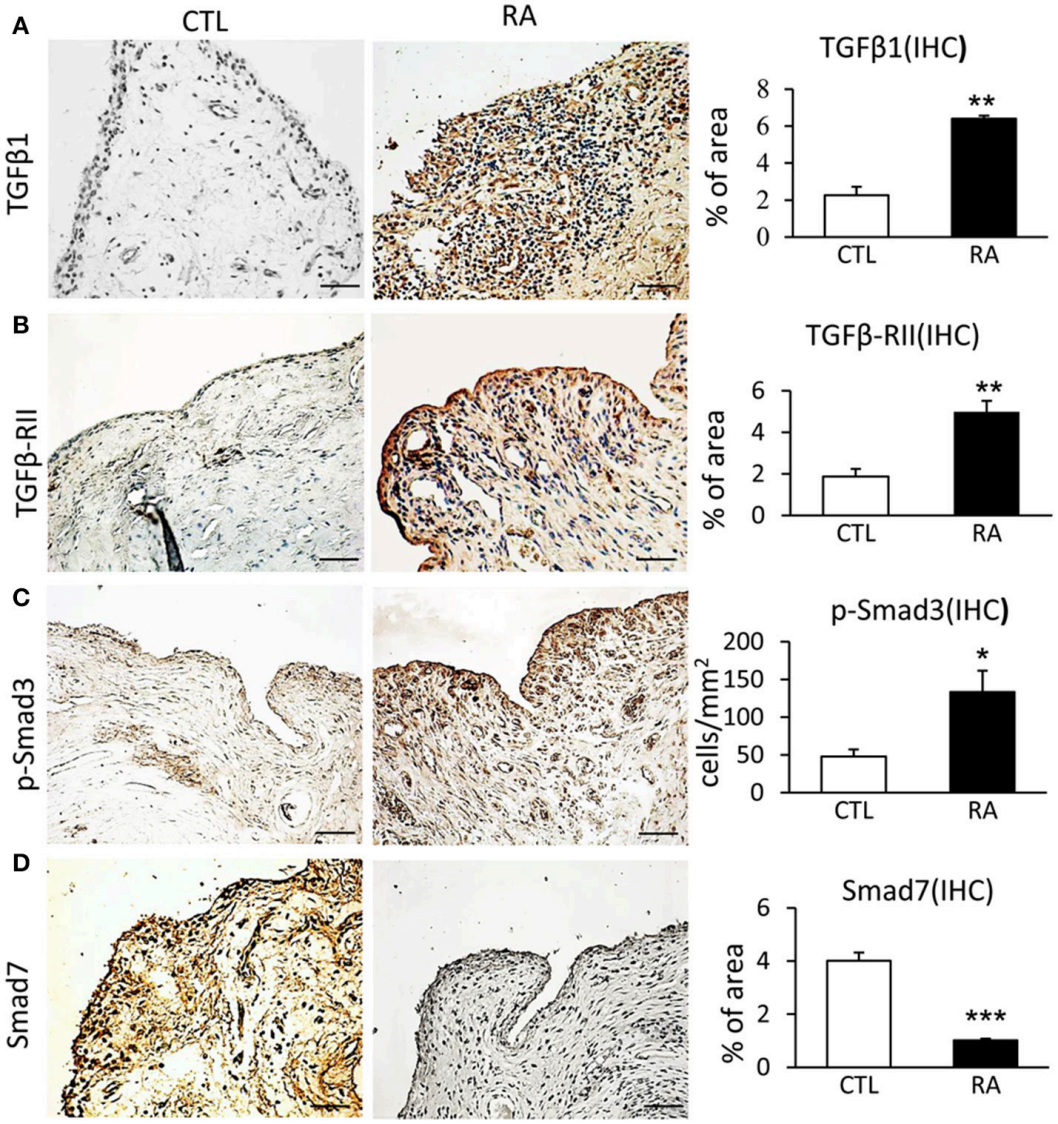
Immunohistochemistry shows an imbalance of TGF-β/Smad signaling with overaction of Smad3 but loss of Smad7 in the synovial tissues of RA patients. **(A)** Expression of TGF-β1. **(B)** Expression of TGF-β receptor II. **(C)** Activation of Smad3 signaling identified by phospho-Smad3 nuclear location. **(D)** Expression of Smad7. Data represent mean ± S.E. for 14 RA patients. *p < 0.05, **p < 0.01, ***p < 0.001 compared to normal controls. Scale bar, 50 μm.

To evaluate TGF-β/Smad signaling in RA, synovial tissues from 14 patients with RA were examined by immunohistochemistry and immunofluorescence. Compared to controls, TGF-β/Smad3 signaling was highly activated in the synovial tissues of RA patients as identified by overexpressing TGF-β1, TGF-β receptor II (TβRII), and phospho-Smad3 nuclear translocation (Figures 1A–C). In contrast, expression of Smad7 was largely reduced in the synovial tissues of RA patients (Figure 1D), demonstrating that the imbalance of TGF-β/Smad signaling occurs in RA tissues and that the loss of Smad7 may promote activation of TGF-β/Smad3 signaling and RA inflammation.

**Figure 2:**
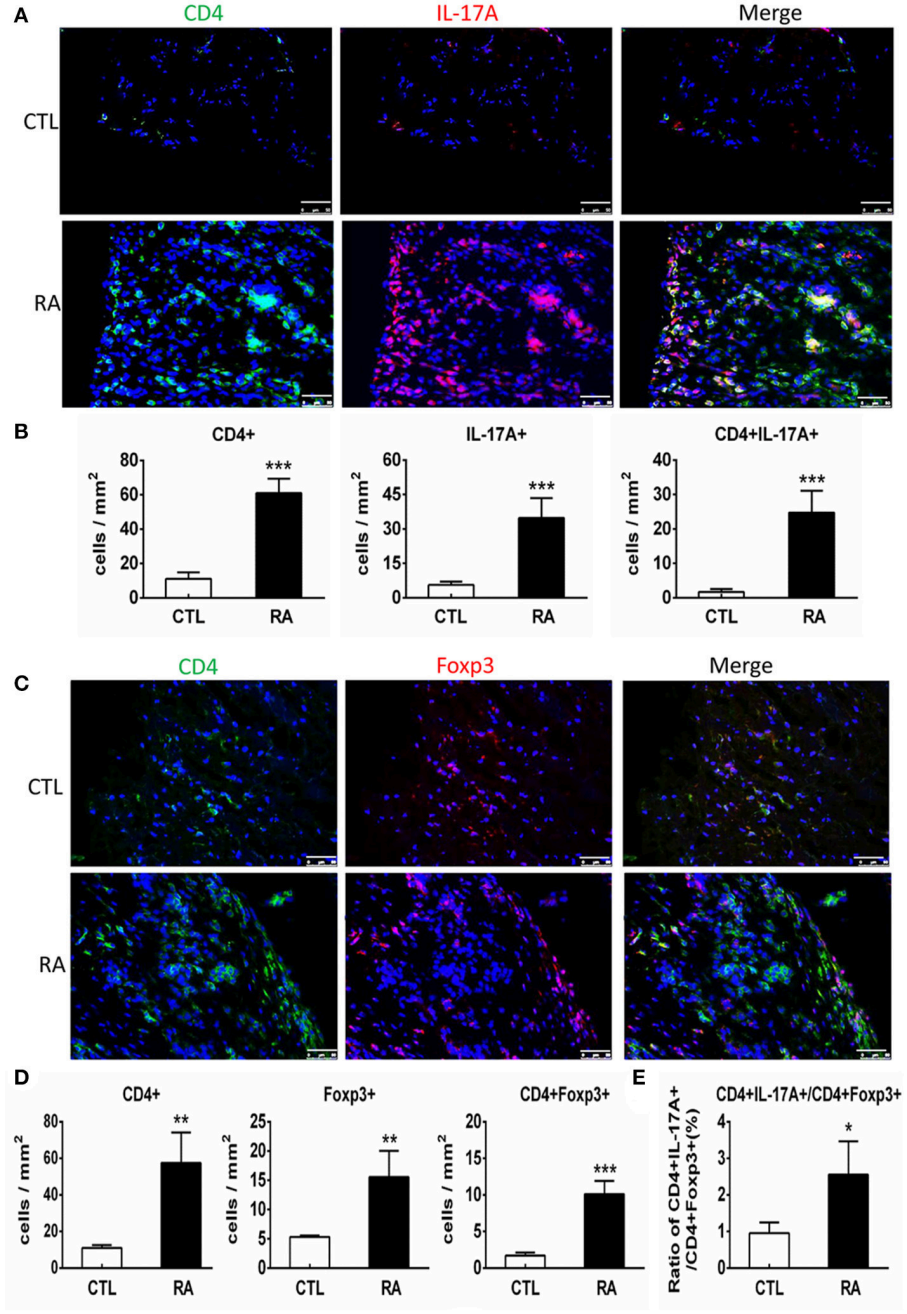
Two-color immunofluorescence detects Th17 and Treg responses in the synovial tissues of RA patients. **(A)** Expression of CD4^+^ IL-17A^+^ cells. **(B)** Quantitative analysis of CD4^+^, IL-17A^+^, and CD4^+^IL-17A^+^ cells. **(C)** Expression of CD4^+^ Foxp3^+^ cells**. (D)** Quantitative analysis of CD4^+^, Foxp3^+^, and CD4^+^ Foxp3^+^ cells.**(E**) Ratio of Th17/Treg cells. Note that there is a 2.7-fold increase in the number of CD4^+^ IL-17A^+^ cells compared to CD4^+^ Foxp3^+^ cells in patients with RA. Data represent mean ± S.E. for 14 RA patients. *p < 0.05, **p < 0.01, ***p < 0.001 compared to normal controls. Scale bar, 50 μm.

We next examined T cell immune response in patients with RA by two-color immunofluorescence. As shown in Figure 2, both CD4^+^ IL-17^+^ cells and CD4^+^ Foxp3^+^ cells increased in the synovial tissues of RA patients. However, the number of CD4^+^ IL-17^+^ cells was far excessive than CD4^+^ Foxp3^+^ cells with a 2.7-fold increase in the synovial tissues of RA patients. This strongly suggests that the imbalance of Th17 over the Treg existed in the synovial tissue of patients with RA. Interestingly, two-color immunofluorescence also detected that patients with RA increased both CD4^+^ IFN-γ^+^ and CD4^+^ IL-4^+^ cells in the synovial tissues with more favorable Th1 response over Th2 (ratio Th1:Th2 = 1.58:1) as shown in Supplementary Figure 1.

### Smad7 deficiency increases susceptibility to autoimmune arthritis in CD-1 mice

**Figure 3:**
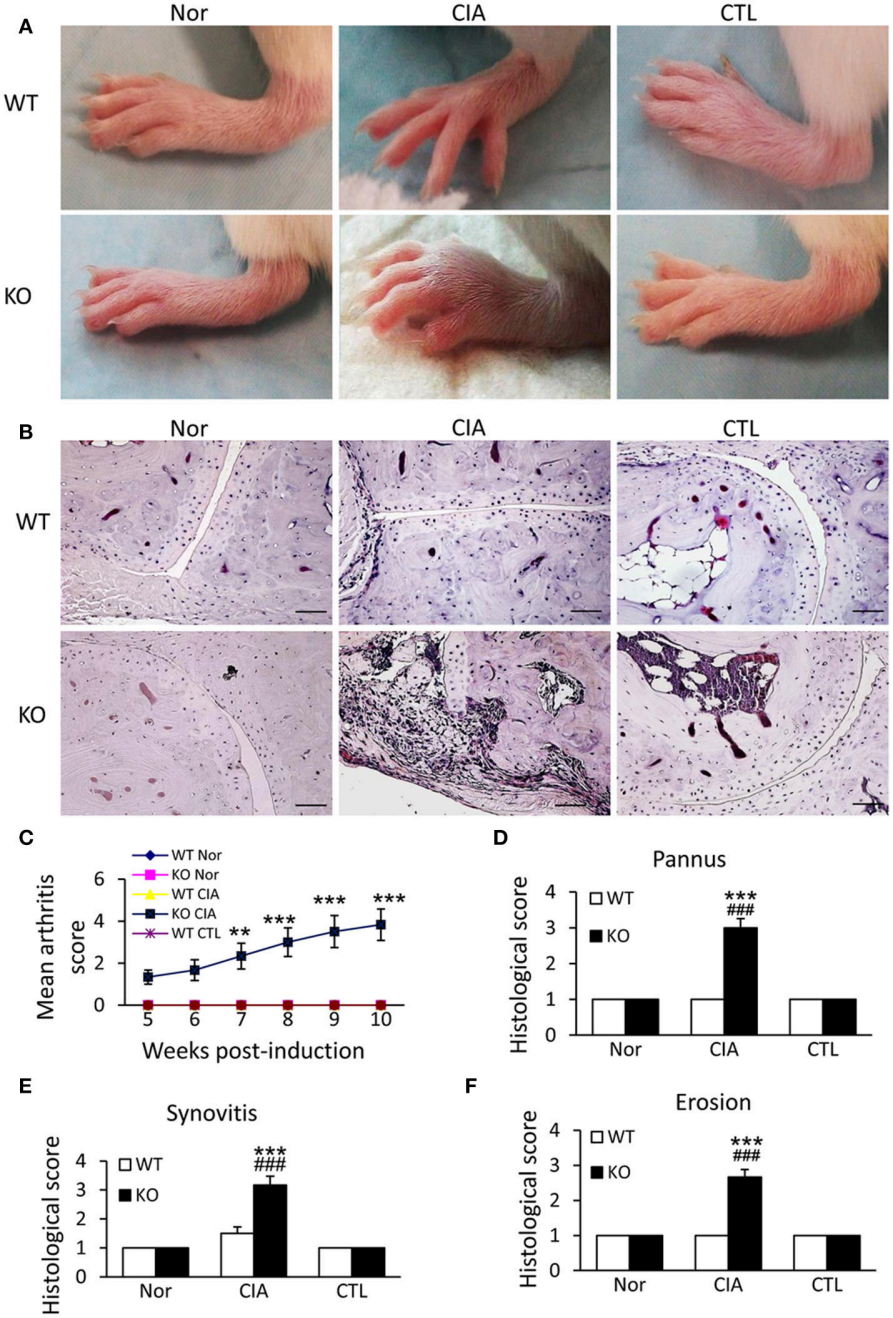
Smad7 deficiency promotes the susceptibility to CIA in CD-1 mice. **(A)** Severe swelling of paws is developed in Smad7-KO but not in Smad7-WT CIA mice at week 10 after disease induction. (**B)** Severe pathological changes with massive inflammatory responses are developed in Smad7-KO but not in Smad7-WT CIA mice at week 10 after disease induction. Scale bar, 50 μm. (**C–F)** Pathological mean scores of arthritis **(C)** pannus **(D)** synovitis **(E)** and erosion **(F)** at week 10 of paws tissues. Data are expressed as mean ± S.E. for group of 8 mice. **p < 0.01, ***p < 0.001 vs. control (CTL); ^*###*^*p* < 0.001 vs. CIA-Smad7-WT mice.

We next examined the potential roles of Smad7 in a mouse model of CIA in Smad7 WT and KO mice (CD1 background). As expected, the CD-1 Smad7 WT mice did not develop any syndromes of RA (Figure 3A and Supplementary Figure 2A), which is consistent with previous reports that the CD-1 mice are resistant to CIA or collagen antibody-induced arthritis(CAIA) (25–28). Surprisingly, four weeks after CIA induction, Smad7-KO CD-1 mice (75%) developed severe CIA as demonstrated by the severe swollen of ankles and toes (Figures 3A,C and Supplementary Figure 2A). This was associated with synovial hyperplasia, inflammatory cell infiltration, pannus emergence, cartilage and bone erosion (Figures 3B,D–F).

### Smad7 deficiency promotes CIA synovial inflammation in CD-1 mice

**Figure 4:**
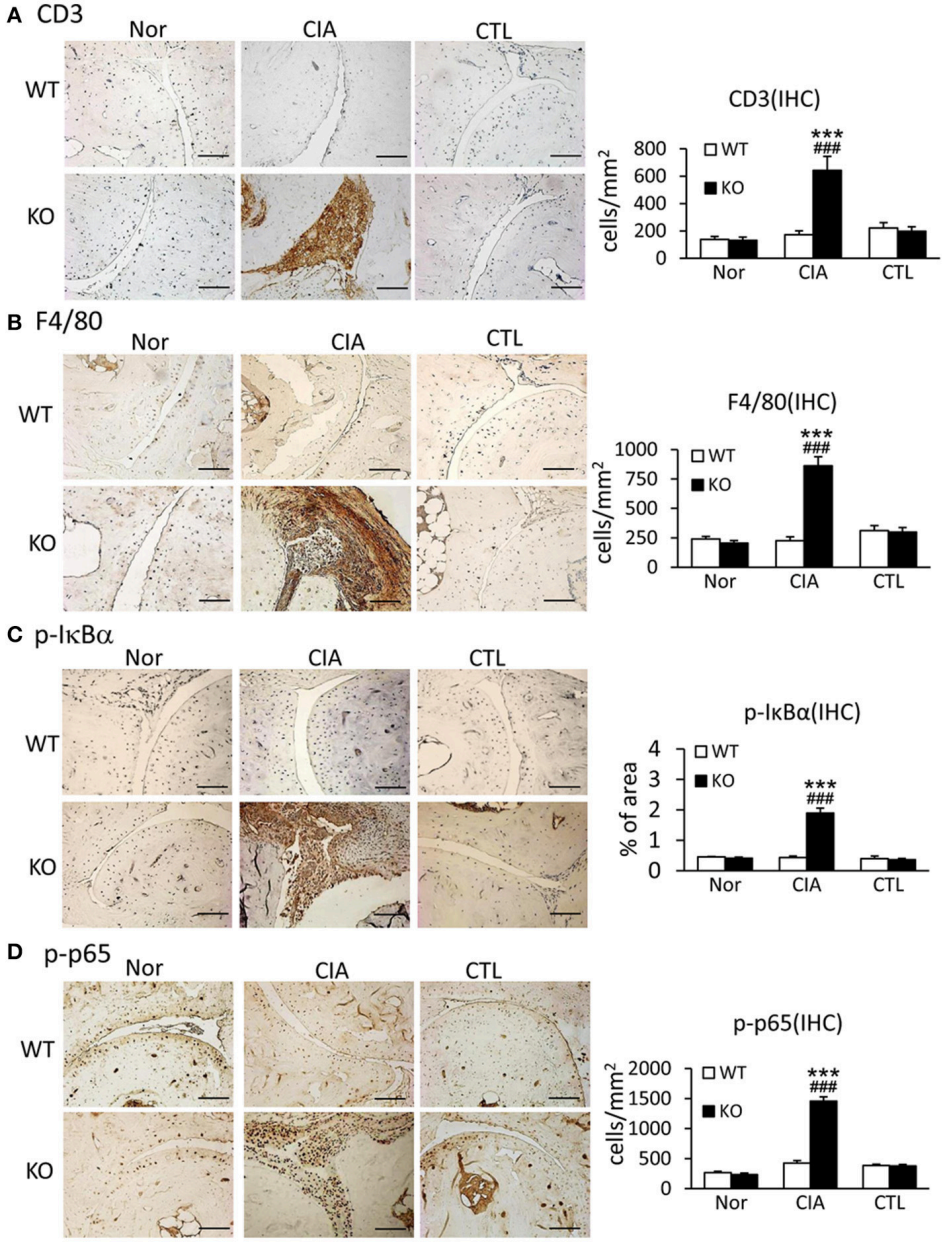
Deletion of Smad7 largely enhances synovial inflammation and activation of NF-κB signaling in CIA mice. **(A)** CD3 expression. **(B**) F4/80 expression. **(C)** Phospho-IκBα. (**D)** Phospho-p65. Results show that severe joint inflammation evidenced by many CD3^+^ T cells and F4/80^+^ macrophages and a marked activation of NF-κB signaling developed in Smad7-KO but not in Smad7-WT CIA mice (CD-1 background). Data are expressed as mean ± S.E. for group of 8 mice. ***p < 0.001 vs. control (CTL); ^*###*^*p* < 0.001 vs. WT-CIA group. Scale bar, 50 μm.

Since uncontrollable inflammation caused by over-activated immune cells and inflammatory cytokines is a critical motivator in the development of RA, we examined whether Smad7 deficiency promotes joint inflammation. Both immunohistochemistry and real-time PCR detected that synovial inflammation was not evidenced in Smad7 WT mice, but largely developed in Smad7 KO CD-1 mice with a marked infiltration of CD3^+^ T cells and F4/80^+^ macrophages, and upregulation of pro-inflammatory cytokines including interleukin-1β (IL-1β), tumor necrosis factor-α (TNF-α), and monocyte chemotactic protein-1 (MCP-1) (Figures 4A,B and Supplementary Figures 2B, 3). Further study demonstrated that enhanced CIA-induced joint inflammation in Smad7 KO mice was associated with a marked activation of NF-κB signaling as detected by remarkable increase in phospho-IκBα and phospho-NF-κB/p65 in the synovial tissues (Figures 4C,D), indicating that the loss of Smad7 causes joint inflammation and NF-κB over-activation.

### Disrupted Smad7 alters the immune-balance by promoting Th17 over treg response in CD-1 mice

**Figure 5:**
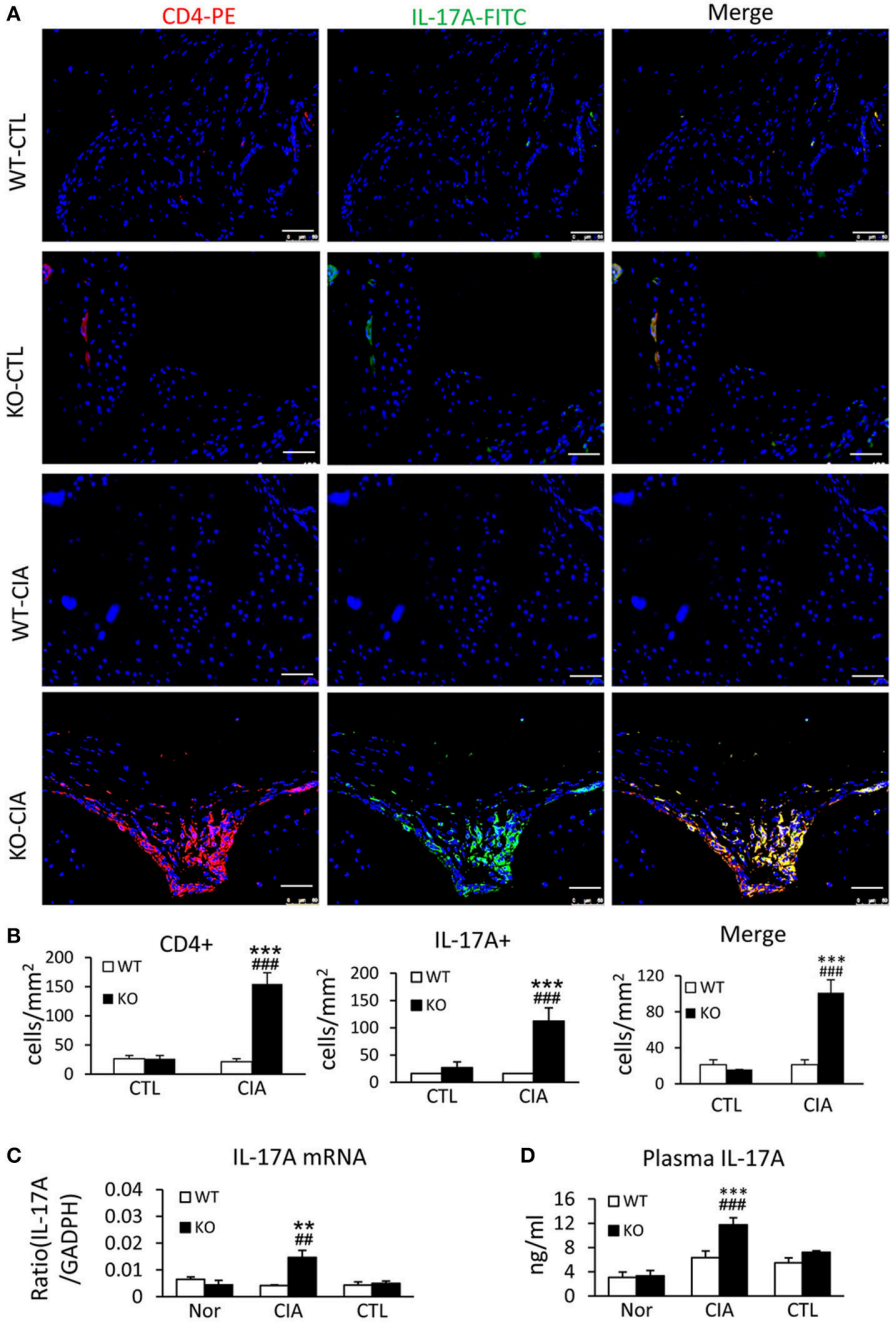
Smad7 deficiency largely enhances the Th17 response both systemically and locally in the synovial tissues of CIA in CD-1 mice. **(A)** Two-color immunofluorescence for detection of CD4^+^ IL-17A^+^ cells in the synovial tissues. **(B)** Quantitative analysis of CD4^+^, IL-17A^+^, and CD4^+^ IL-17A^+^ cells. **(C)** Real-time PCR analysis of IL-17A mRNA expression. **(D)** ELISA analysis of serum levels of IL-17A. Results show a marked Th17 response developed in Smad7-KO but not in Smad7-WT mice both systemically and locally in the joint tissues after disease induction at week 10. Data are expressed as mean ± S.E. for group of 8 mice. **p < 0.01, ***p < 0.001 vs. control group; ^*##*^*p* < 0.01, ^*###*^*p* < 0.001 vs. WT-CIA group. Scale bar, 50 μm.

It has been shown that enhanced Th17 response is a key mechanism of RA (29, 30). We next examined whether loss of Smad7 promotes Th17 response in CIA. As shown in Figures 5A,B deletion of Smad7 resulted in a marked increase in CD4^+^IL-17A^+^ cells in the synovial tissues in response to collagen II injection, whereas no Th17 response was evidenced in Smad7 WT mice. Real-time PCR and ELISA also detected that Smad7 KO mice exhibited a marked upregulation of IL-17 mRNA in the diseased joint tissues with elevated plasma levels of IL-17A (Figures 5C,D), demonstrating that the loss of Smad7 triggers the Th17 response in CIA.

**Figure 6:**
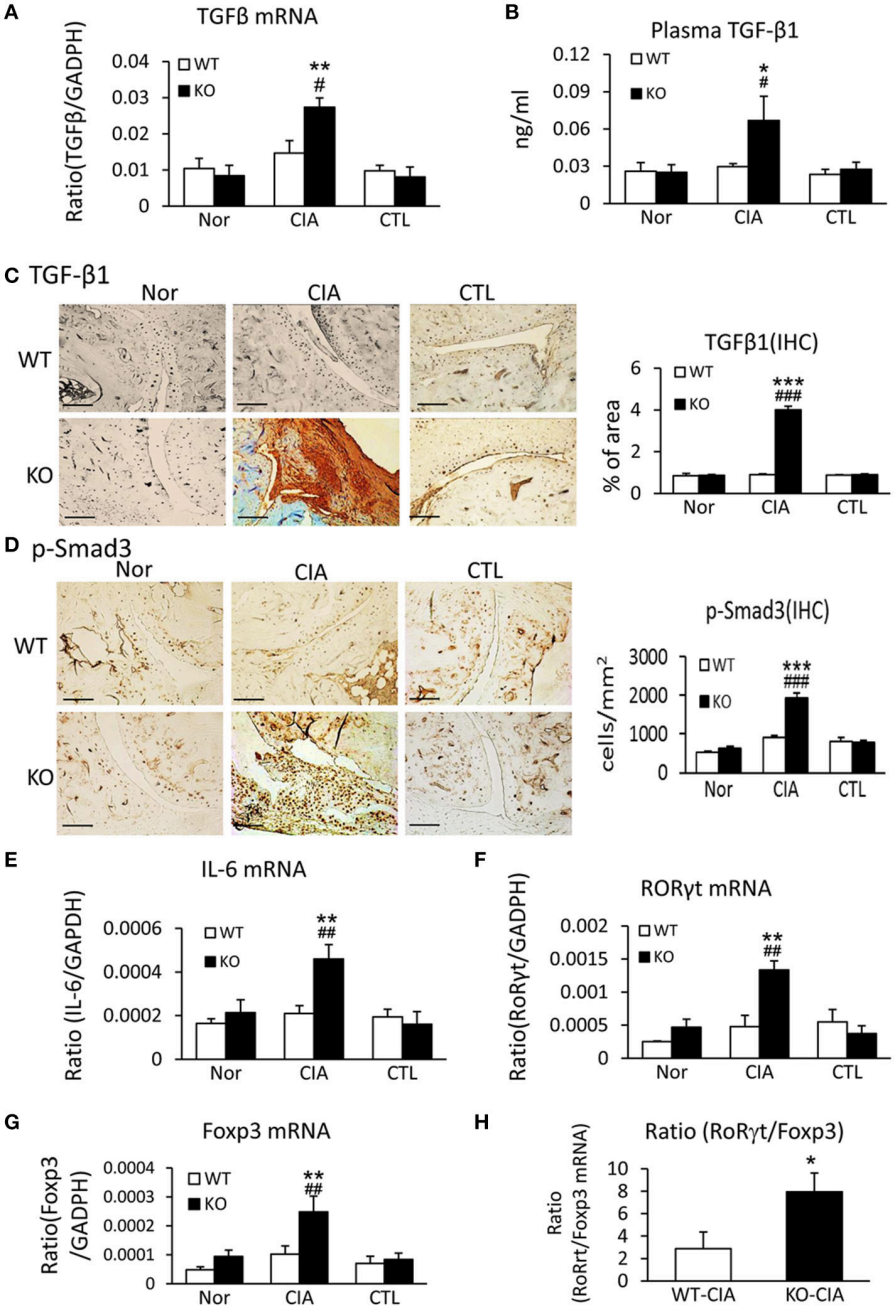
Deletion of Smad7 enhances TGF-β1/Smad3 and IL-6 signaling and promotes Th17 over the Treg responsein the synovial tissues of CIA in CD-1 mice. **(A)** Synovial TGF-β1 mRNA expression by real-time PCR. **(B)** Plasmalevels of TGF-β1 by ELISA. **(C)** Synovial IL-6 mRNA expression by real-time PCR. **(D)** Synovial RORγt mRNA expression by real-time PCR. **(E)** Synovial TGF-β1 expression by immunohistochemistry. **(F)** Activation of Smad3 signaling identified by phospho-Smad3 (p-Smad3) nuclear translocation. **(G)** Synovial Foxp3 mRNA expression by real-time PCR. **(H)** Ratio of synovial RORγt/Foxp3 mRNA expression. Data are expressed as mean ± S.E. for group of 8 mice. *p < 0.05, **p < 0.01, ***p < 0.001 vs. control (CTL); ^#^*p* < 0.05, ^*##*^*p* < 0.01, ^*###*^*p* < 0.001 vs. WT-CIA group. Scale bar, 50 μm.

We next investigated the potential mechanisms by which deletion of Smad7 promotes Th17 response by studying both TGF-β and IL-6 signaling in the synovial tissues, since TGF-β1, together with IL-6, can promote Th17 response via the orphan nuclear receptor retinoid-related orphan receptor (ROR) γt-dependent mechanism (31, 32). As shown in Figures 6A–D, real-time PCR, immunohistochemistry, and ELISA detected that deletion of Smad7 largely increased TGF-β1 expression both locally in synovial tissues and systemically in plasma, which resulted in the activation of Smad3 signaling as evidenced by a marked increase in phospho-Smad3 nuclear translocation in the diseased joint tissues. Interestingly, real-time PCR also detected that deletion of Smad7 upregulated IL-6 and RORγt mRNA expression in synovial tissues of CIA in CD-1 mice, but not in Smad7 WT-CIA mice (Figures 6E,F). These findings suggest that loss of Smad7 promotes Th17-mediated autoimmune arthritis in CD-1 mice, a mouse strain resistant to the development of CIA.

**Figure 7:**
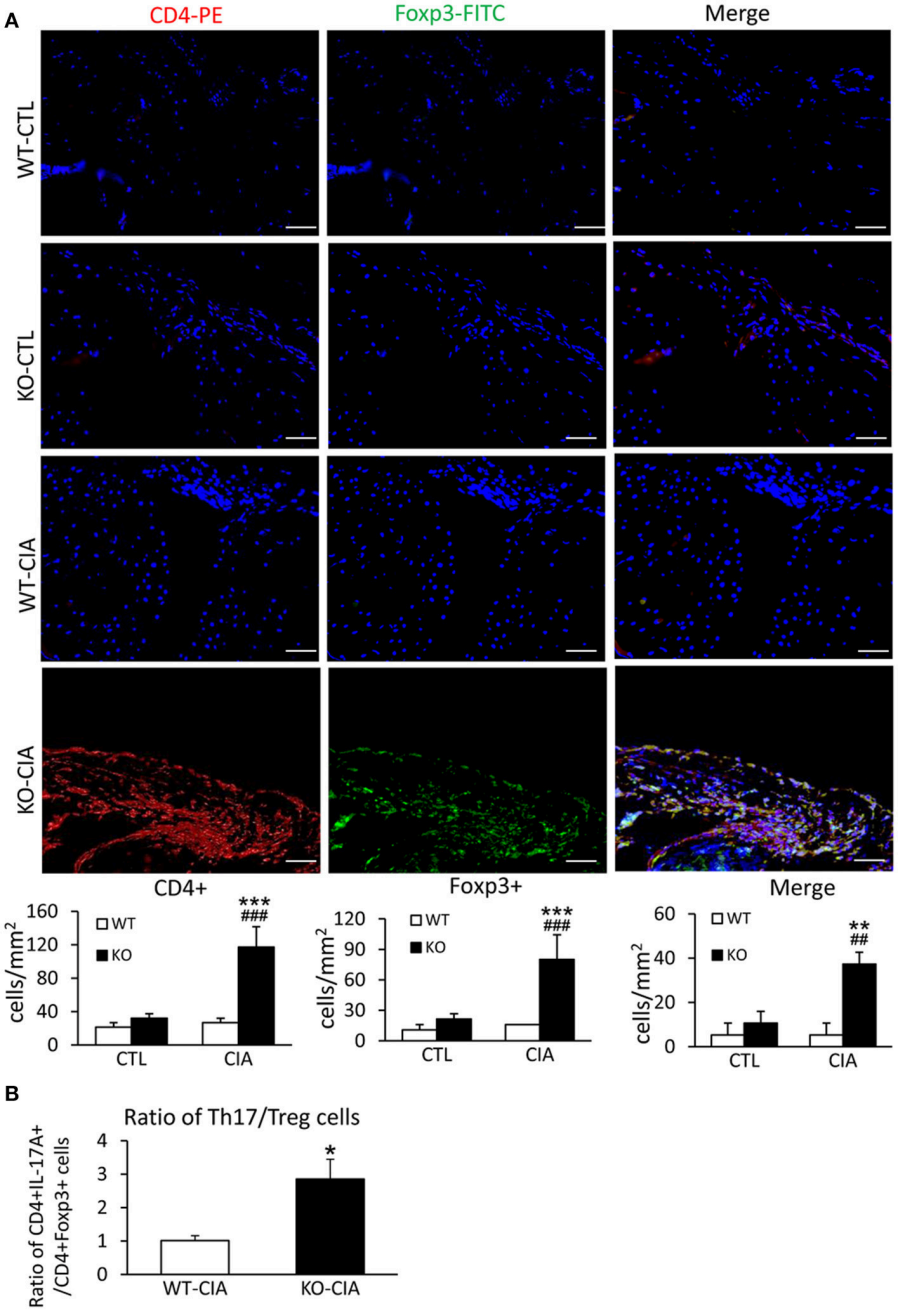
Deletion of Smad7 increases Treg cells but causes imbalance of Th17 over Treg response with the high ratio of CD4^+^ IL-17A^+^/CD4^+^ Foxp3^+^ cells in the synovial tissues of CIA in CD-1 mice. **(A)** Two-color immunofluorescence of CD4^+^Foxp3^+^ cells in synovial tissues in CD-1 mice. **(B)** The ratio of CD4^+^ IL-17A^+^ cells/ CD4^+^ Foxp3^+^ cells in synovial tissues of CIA mice. Data are expressed as mean ± S.E. for group of 8 mice. *p < 0.05, **p < 0.01, ***p < 0.001 vs. control (CTL); ^*##*^*p* < 0.01, and ^*###*^*p* < 0.001 vs. CIA-Smad7-WT mice. Scale bar, 50 μm.

**Figure 8:**
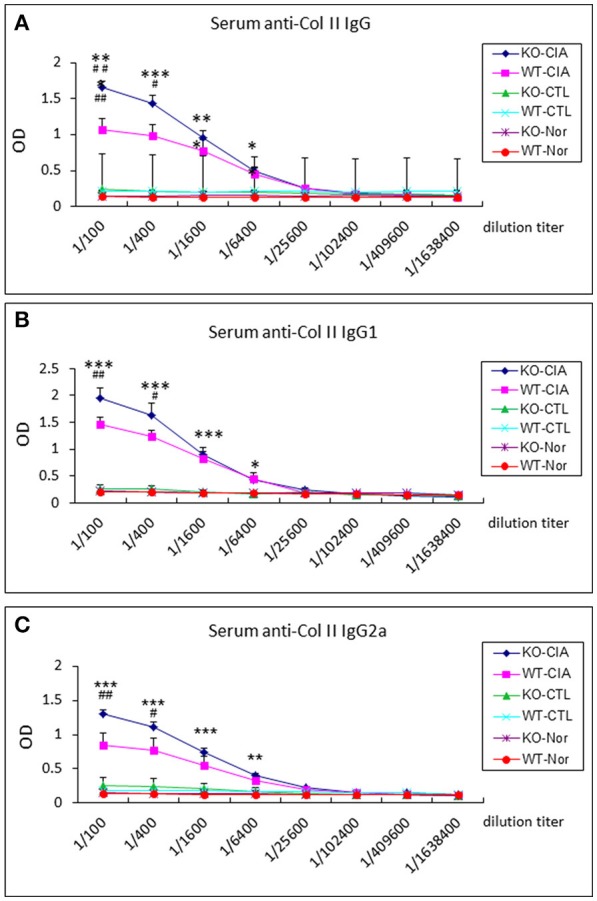
Deletion of Smad7 increases serum levels of anti-collagen II IgG subtypes in CIA mice by ELISA. Note that deletion of Smad7 significantly increases serum levels of anti-collagen II total IgG **(A)**, IgG1 (Th2, **(B)**) and IgG2a (Th1, **(C)**) in CIA mice (CD-1). Data represent mean ± S.E. for groups of 8 mice. *p < 0.05, **p < 0.01, ***p < 0.001 compared to control; ^#^*p* < 0.05, ^*##*^*p* < 0.01 compared to CIA-WT mice.

It is also known that the activation of TGF-β/Smad3 signaling can promote Treg response via a Smad3-dependent mechanism. Indeed, real-time PCR showed that deletion of Smad7 upregulated Foxp3 transcription (Figure 6G) and thus increased CD4^+^Foxp3^+^Treg cells in the diseased synovial tissues (Figure 7A), suggesting an increased Treg response in Smad7 KO CIA-mice. However, by comparison analysis it clearly demonstrated that disrupted Smad7 significantly altered the balance between Th17 and Treg by a 2.7-fold increase in the ratio of RORγt/Foxp3 mRNA (Figure 6H) and a 3-fold increase in the ratio of CD4^+^ IL-17A^+^/CD4^+^ Foxp3^+^ cells (Figure 7B). Interestingly, deletion of Smad7 promoted an equal Th1 and Th2 immune response locally in the diseased tissues by increasing both Th1 and Th2 master transcriptional factor (T-bet/GATA3) and their signature cytokines IFN-γ and IL-4 (Supplementary Figure 4) without altering the ratio of CD4^+^ IFN-γ^+^ cells/CD4^+^ IL-4^+^ cells (Supplementary Figures 5, 6). This was also supported by the ELISA finding that deletion of Smad7 resulted in a significant increase of anti-collagen II total IgG and subclasses of IgG1 (Th2-dependent) and IgG2a (Th1-dependent) when compared to Smad7 WT CIA mice (Figure 8). Taken together, these novel findings suggest that disrupted Smad7 increases the susceptibility to CIA by enhancing both Th1 and Th2 response and importantly, by triggering the Th17 over the Treg immune response.

## Discussion

In the present study, we reported that Smad7 plays a protective role in CIA. This was supported by the finding that loss of Smad7 was associated with a marked activation of TGF-β/Smad3 signaling and the development of arthritis in patients with RA. Surprisingly, disruption of Smad7 from CD-1 mice, a strain of CIA resistance, greatly promoted the development of autoimmune arthritis including severe swollen of ankles and toes with synovial hyperplasia, massive inflammatory cells infiltration, pannus emergence, and cartilage/bone erosion. Findings from this study revealed a critical role of TGF-β/Smad signaling in the pathogenesis of autoimmune arthritis and identified Smad7 as a negative regulator of autoimmune arthritis.

It is well known that Smad7 is an important inhibitor in the feedback-loop of TGF-β/Smad signaling and also functions as an integrator, not only to block TGF-β/Smad but also to inhibit NF-κB signaling by inducing a NF-κB inhibitor IKBα (16, 33–35). Thus, disruption of Smad7 not only promotes TGF-β/Smad3 signaling and tissue fibrosis, but also largely enhances NF-κB-driven inflammation in several disease models (36–38). Consistent with the protective role of Smad7 in tissue fibrosis and inflammation, we found that loss of Smad7 largely enhanced activation of both TGF-β/Smad3 and NF-κB signaling in the synovial tissues in response to CIA. This may well explain why deletion of Smad7 promoted autoimmune arthritis in CD-1 mice. In contrast, intraarticular overexpression of Smad7 ameliorates experimental arthritis (39). Thus, Smad7 may function to balance the TGF-β/Smad and NF-κB signaling and hence determines the occurrence and the severity of CIA, as illustrated in this study.

Th17/Treg imbalance has been reported to contribute to a variety of inflammatory autoimmune diseases (29, 40–42). It is reported that active RA patients exhibited hyper-reactive Th17 while decreasing Treg response (40). Interestingly, the present study detected that disruption of Smad7 significantly increased Th1, Th2, Th17, and Treg immune responses and production of antigen-specific antibodies including anti-collagen II IgG and subclasses of IgG1 and IgG2a in CIA mice, demonstrating a negatively regulatory role of Smad7 in T cell immunity in response to CIA. It has been reported that Th17 is an effective B cell helper (43) and IL17 has been show to play a regulatory role in antibody production (44). In the present study, we also found that a significant increase in Th17 cells in Smad7 KO mice was accompanied with higher levels of anti-collagen antibodies. It is possible that disrupted Smad7 increased TGF-β signaling and thus promoted Th17-dependent B cell activation and anti-collagen II antibody production as seen in Smad7 KO mice with CIA. In addition, follicular helper T (Tfh) cell is critical for GC formation and antibody production (45) and previous studies showed that active TGF-β signaling could promote Tfh cell differentiation (46). In this study, we showed that deletion of Smad7 promoted TGF-β/Smad3 signaling, which might stimulate the Tfh differentiation and autoantibody production. Thus, it is likely that Smad7 defection may result in autoantibody overproduction via both Th17 and Tfh-dependent mechanisms.

Further studies by using ratio analysis showed that while disruption of Smad7 promoted both Th1 and Th2 equally, excessive Th17 over Treg responses were detected in arthritic joint tissues. Specifically, the intensity of Th17 response was double of that by Treg as evidenced at the levels of transcription (RORγt > Foxp3), cytokines (IL-17A > IL-4), and infiltrating cells (CD4+ IL-17A+> CD4+ Foxp3+ cells). These observations further revealed that disruption of Smad7 not only promotes T cell immune responses in general, but also causes the imbalance of Th17 over the Treg immune response. It is well documented that TGF-β signaling promotes the differentiation of Treg cells via a Foxp3-dependent mechanism (47), while inducing Th17 differentiation in combination with IL-6 via the RORγt-dependent pathway in patients with RA (48, 49). In this study, we found that Smad7 deficiency largely enhanced TGF-β signaling and IL-6 expression, which may be responsible for the upregulation of RORγt and an increase in Th17 differentiation. IL-17 may mediate tissue inflammation by inducing other pro-inflammatory cytokines (such as IL-6, IL-1β, and TNF-α), which in turn may activate the downstream pathways including NF-κB to further amplify the inflammatory cascade (50–52). By this means, the pro-inflammatory effects of Th17 may override the protective effects of Treg cells. Therefore, increased TGF-β/Smad3 and IL-6 signaling may be a key mechanism through which disruption of Smad7 promotes RORγt-mediated Th17-dependent autoimmune arthritis as seen in this study.

In conclusion, the present study revealed that Smad7 deficiency promoted autoimmune arthritis and enhanced NF-κB activation, Th1/Th17 differentiation and synovial inflammation, which might result from the uncontrollable TGF-β/Smad3-IL-6 signaling due to Smad7 defection. Results from this study suggest that Smad7 may be a novel therapeutic agent for treatment of autoimmune arthritis.

## Author contributions

All authors listed have made a substantial, direct and intellectual contribution to the work, and approved it for publication.

### Conflict of interest statement

The authors declare that the research was conducted in the absence of any commercial or financial relationships that could be construed as a potential conflict of interest.
