# Supplementary material for: Loss of Smad7 Promotes Inflammation in Rheumatoid Arthritis
Source: Front Immunol. 2018 Nov 2;9:2537. doi: 10.3389/fimmu.2018.02537 (PMC6224447; doi:10.3389/fimmu.2018.02537)
Supplement: Supplementary file 1 [file Data_Sheet_1.PDF]

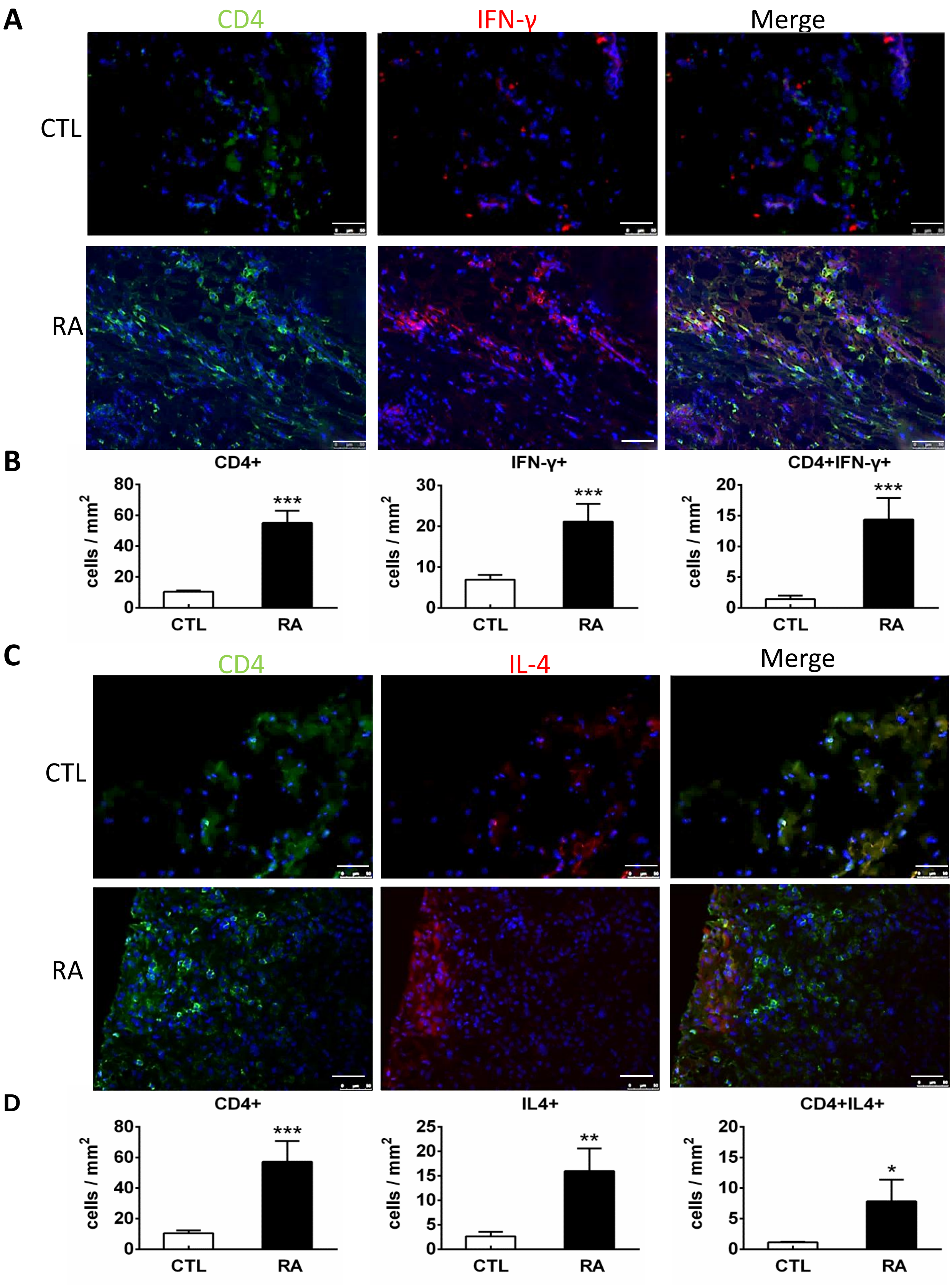

Suppl Figure 1. Two-color immunofluorescence detects Th1 and Th2 responses in synovial tissues of patients with RA. A and B. CD4+IFNγ+ cells. **B and C.** CD4+IL-4+ cells. Note that there are more CD4+IFN-γ+ cells than CD4+IL-4+ cells in RA patients. Data represent mean ± S.E. for 14 RA patients. . *p<0.05, ***p<0.001, compared to control. Scale bar, 50 µm.

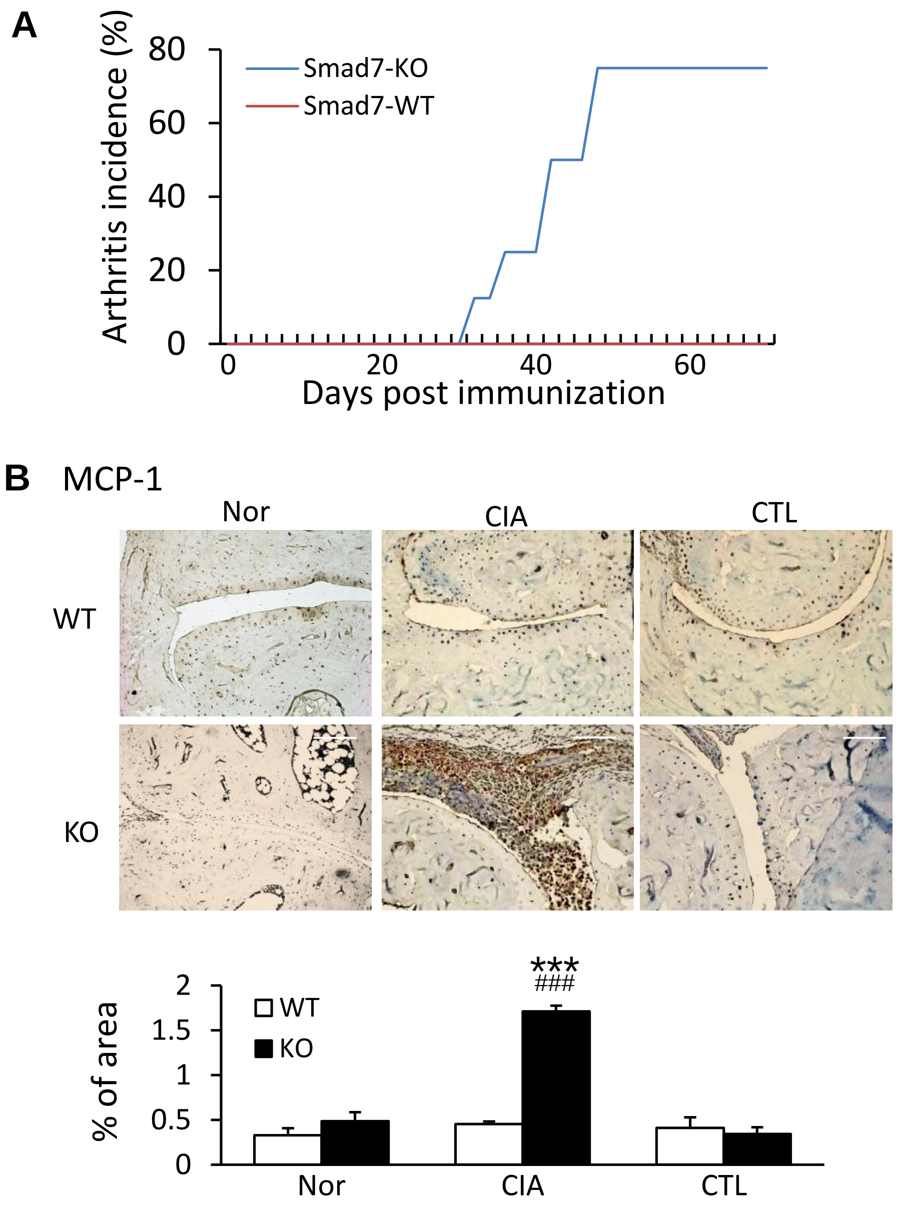

Suppl Figure 2. Deletion of Smad7 largely promotes CIA in CD-1 mice. A. Incidence rat of arthritis. B. Immunohistochemistry of MCP-1 expression. Data represent mean±S.E. for groups of 8 mice. ***p<0.001 compared to control; ###p<0.001 compared to WT CIA-mice. Scale bar, 50 μM.

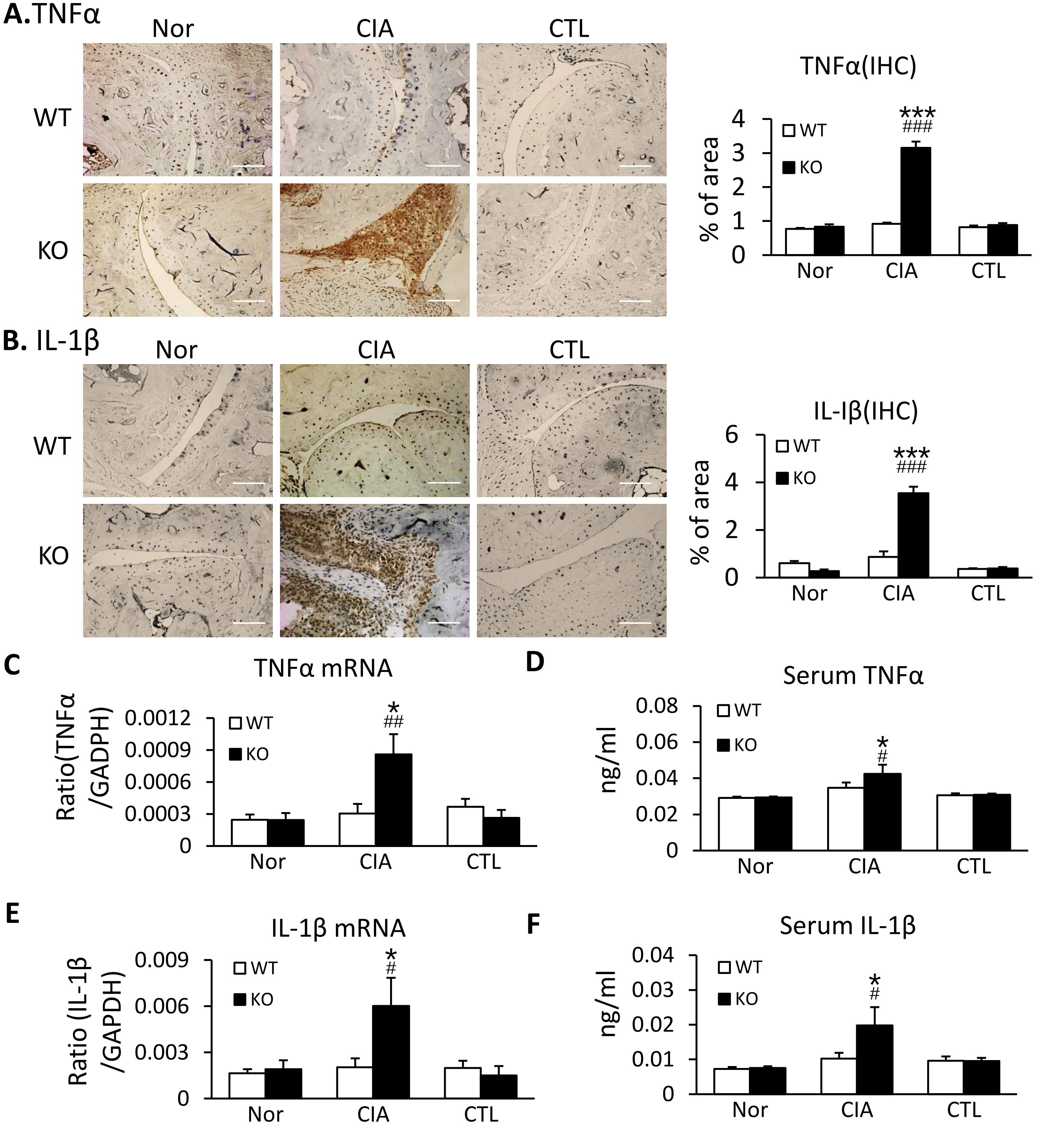

Suppl Figure 3. Deletion of Smad7 largely upregulates pro-inflammatory cytokines IL-1β and TNFα in synovial tissues of CIA in CD-1 mice. A and B. Immunohistochemistry of IL-1β and TNFα expression. **C and D.** Real-time PCR and ELISA detection of TNFα in joint tissues and serum. **E and F.** Real-time PCR and ELISA detection of IL-1β in joint tissues and serum. Data represent mean ± S.E. for groups of 8 mice. *p<0.05, ***p<0.001, compared to control; #p<0.05, ##p<001, ###p<0.001 compared to WT CIA-mice. Scale bar, 50 μM.

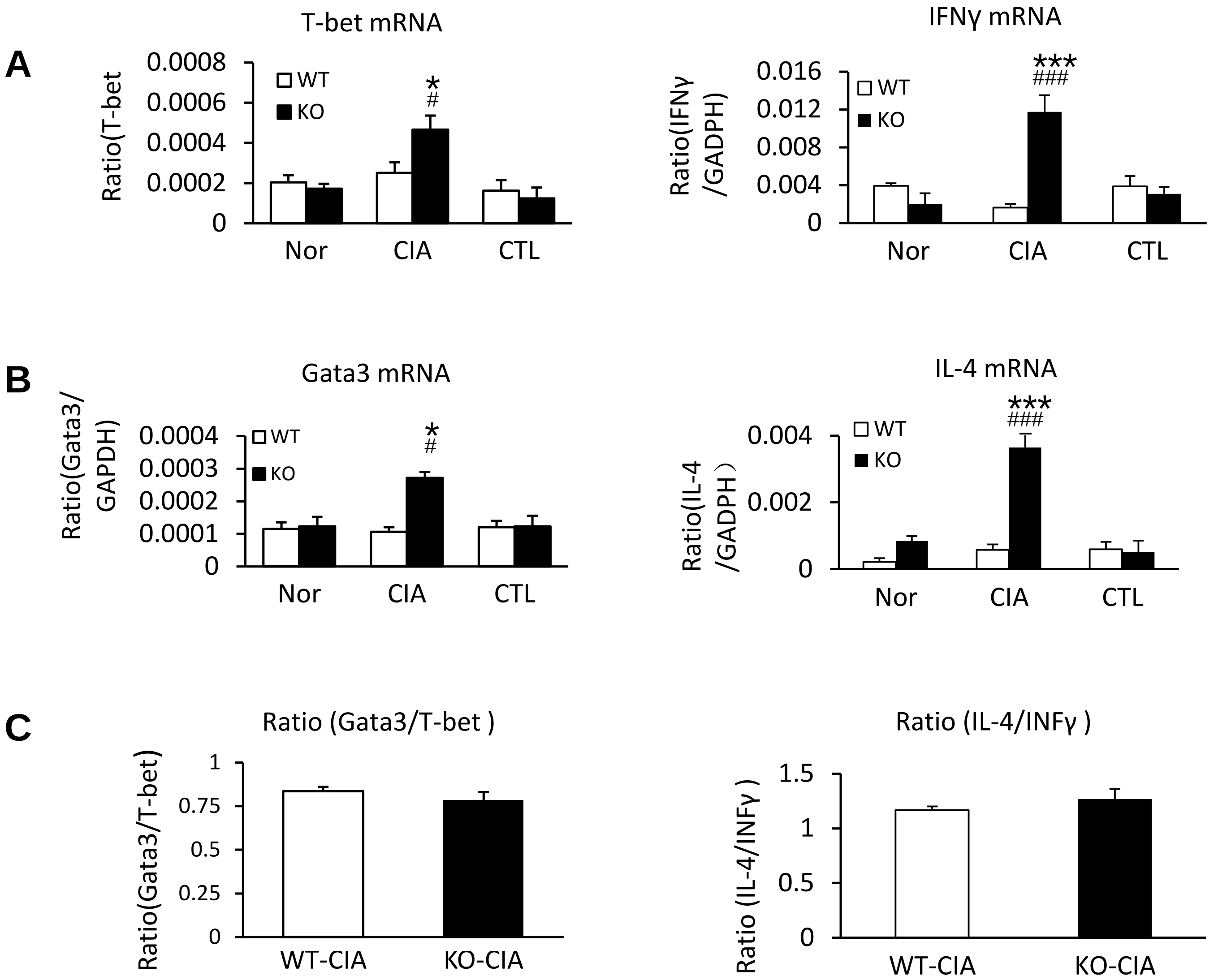

Suppl Figure 4. Real-time PCR shows that deletion of Smad7 upregulates T-bet /INF γ and Gata3 /IL-4mRNA in synovial tissues of CIA in CD-1 mice. A. T bet /INF γ mRNA**. B.** Gata3/IL-4 mRNA. **C.** Ratio of Gata3/T bet and IL-4/INFγ). Note that no difference in the ratio of Gata3/T-bet and IL-4/INFγ is found between Smad7 WT and KO mice with CIA. Data represent mean±S.E. for groups of 8 mice. *p<0.05 compared to control; ##p<001compared to WT CIA-mice.

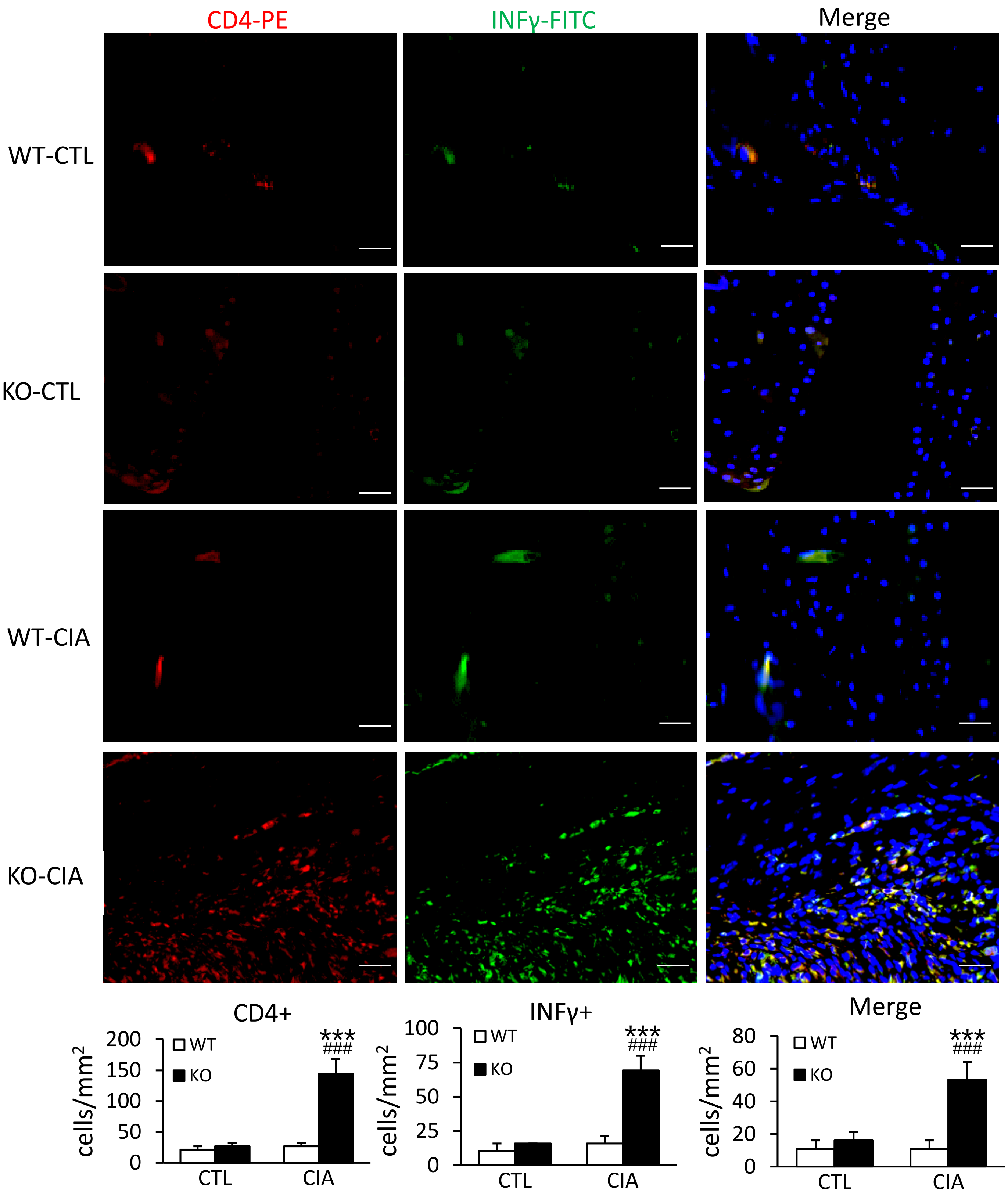

Suppl Figure 5. Two-color immunofluorescence detects that deletion of Smad7 increases Th1 responses (CD4+INFγ+ cells) **in synovial tissues of CIA in CD-1 mice.** Data represent mean ± S.E. for groups of 8 mice. ***p<0.001 compared to control; ###p<0.001 compared to CIA-WT mice. Scale bar, 50 µm.

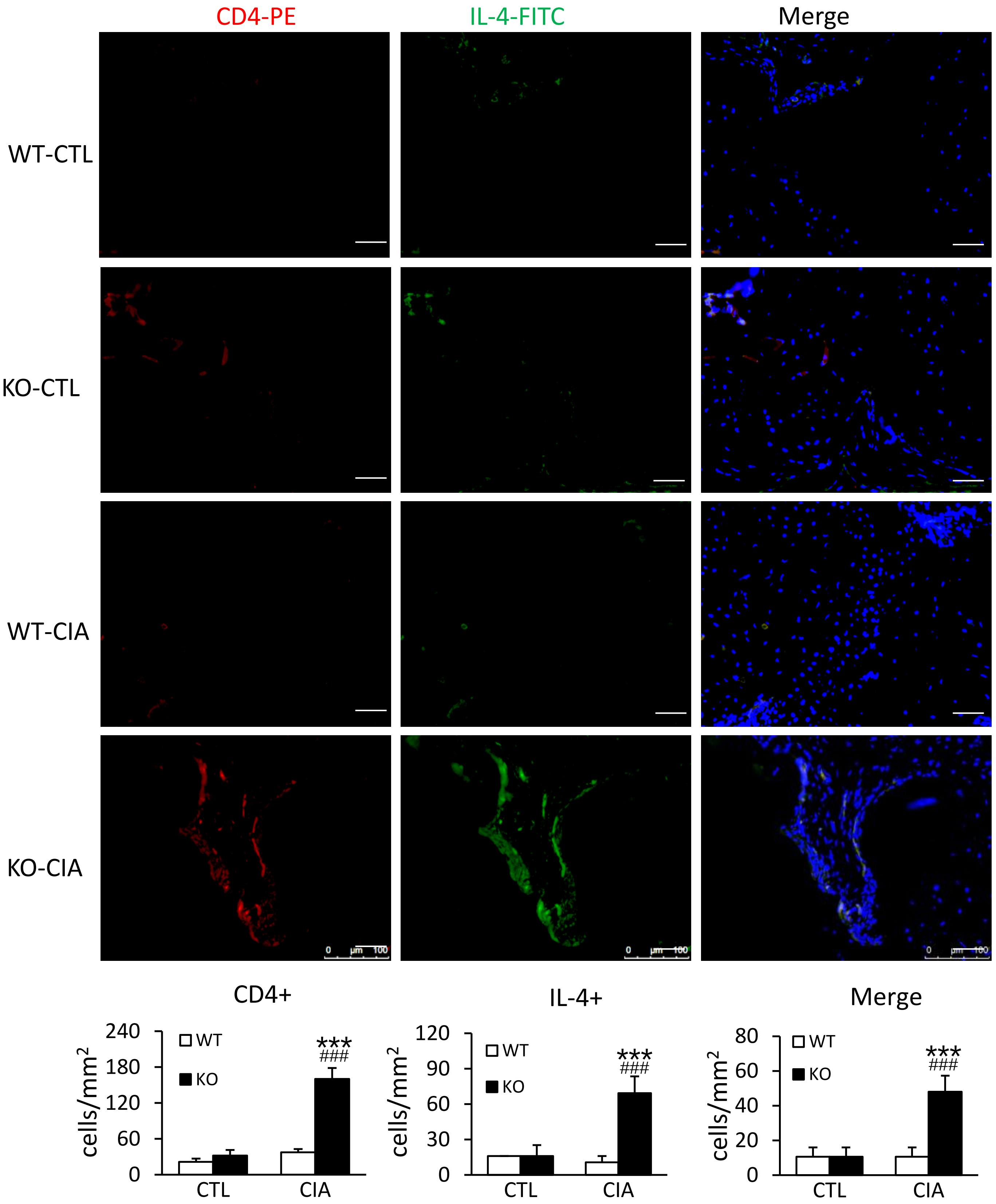

Suppl Figure 6. Two-color immunofluorescence detects that deletion of Smad7 increases Th2 responses (CD4+IL-4+ cells) in synovial tissues of CIA in CD-1 mice. Data represent mean ± S.E. for groups of 8 mice. ***p<0.001 compared to control; ###p<0.001 compared to CIA-WT mice. Scale bar, 50 μm.
